# Validation of the Metabolite Ergothioneine as a Forensic Marker in Bloodstains

**DOI:** 10.3390/molecules27248885

**Published:** 2022-12-14

**Authors:** Seungyeon Lee, Sora Mun, You-Rim Lee, Jiyeong Lee, Hee-Gyoo Kang

**Affiliations:** 1 Department of Senior Healthcare, Graduate School, Eulji University, Uijeongbu 11759, Republic of Korea; 2 Department of Biomedical Laboratory Science, College of Health Sciences, Eulji University, Seongnam 13135, Republic of Korea; 3 Department of Biomedical Laboratory Science, College of Health Science, Eulji University, Uijeongbu 11759, Republic of Korea

**Keywords:** bloodstain, ergothioneine, forensic marker, metabolite, liquid chromatography–tandem mass spectrometry

## Abstract

Ergothioneine, which is a naturally occurring metabolite, generally accumulates in tissues and cells subjected to oxidative stress, owing to its structural stability at physiological pH; therefore, it has been attracting attention in various biomedical fields. Ergothioneine has also been suggested as a potential forensic marker, but its applicability has not yet been quantitatively validated. In this study, quantitative analysis of ergothioneine in bloodstains was conducted to estimate the age of bloodstains and that of bloodstain donors. Blood from youth and elderly participants was used to generate bloodstains. After extracting metabolites from the bloodstains under prevalent age conditions, ergothioneine levels were quantified by mass spectrometry via multiple reaction monitoring. The concentration of ergothioneine in day 0 bloodstains (fresh blood), was significantly higher in the elderly group than in the youth group, but it did not differ by sex. Statistically significant differences were observed between the samples from the two age groups on days 0, 5 and 7, and on days 2 and 3 compared with day 0. The findings suggest that ergothioneine can be used to estimate the age of bloodstains and of the donor; it could be useful as a potential marker in reconstructing crime scenes.

## 1. Introduction

Metabolites are commonly targeted substances for drug analysis, mainly in the field of forensic science. In addition to analyzing drug metabolites in biological samples, some studies have also attempted to examine them in bloodstains to reconstruct crime scenes. By associating time with several measurable variables, it is possible to analyze the age of bloodstains. Furthermore, information on how much time has elapsed since blood was spilled and bloodstains were formed can be useful for deducing the time of occurrence of an event [1]. 

To date, target biomolecules in bloodstains, such as metabolites, hemoglobin, RNA, amino acids and proteins, have been analyzed to estimate the age of bloodstains using high-performance liquid chromatography (HPLC) [2], gas chromatography (GC) [3], mass spectrometry (MS) [4], infrared (IR) spectroscopy [5,6,7], Raman spectroscopy [8,9], electron paramagnetic resonance (EPR) [10,11], hyperspectral spectroscopy [12,13], atomic force microscopy (AFM) [14], reflectance spectroscopy [15,16,17], spectrofluorometry [18,19], polymerase chain reaction (PCR) [20,21] and even smart phones [22,23]. Morphological changes in bloodstains or biochemical changes in bloodstain components can be analyzed using either nondestructive or destructive methods [24,25]. Among these techniques, MS, which is capable of high sensitivity in qualitative and quantitative analyses, is typically used for metabolite analysis of complex bloodstain samples [26]. Hereinafter, MS is used for the identification of metabolites through metabolic profiling of the bloodstain. In addition to the discovery of metabolites to estimate the age of bloodstains [4], some studies have also identified metabolites that can be used as internal standards related to bloodstain volume [27] and metabolite markers to estimate the age of the bloodstain donor [28]. These studies have expanded the selection of blood metabolites available for analysis in forensic science.

According to a previous study on the estimation of the age of bloodstains, the metabolite ergothioneine showed a continuous decrease as time elapsed from day 0 to day 21, suggesting the possibility that ergothioneine could be used for distinguishing bloodstains older than 21 day [4]. In addition, ergothioneine has been discovered as an age marker for bloodstain donors. Its concentration in bloodstain samples from elderly and youth adults showed a marked difference when the samples were stored at room temperature (22 ± 1 °C) and 65 ± 12% relative humidity for up to 4 h [28]. Ergothioneine is a compound that has attracted attention in various fields of medical biology because it accumulates in tissues that are subjected to oxidative stress, such as red blood cells (RBCs), and is structurally stable at physiological pH [29,30]. Previous studies have suggested the use of ergothioneine as a forensic marker can provide useful information for reconstructing crime scenes [28]. However, no study has quantitatively verified the potential role of the ergothioneine present in bloodstains in forensic medicine. Therefore, in this study, quantitative analysis of ergothioneine in bloodstains was conducted to confirm the applicability of ergothioneine as a forensic marker for estimating the age of bloodstains and that of bloodstain donors. 

## 2. Results

### 2.1. Concentration of Ergothioneine in Fresh Blood

**Figure 1:**
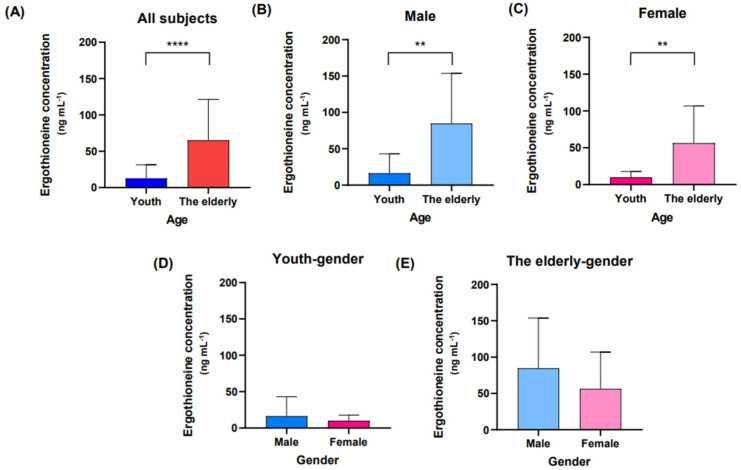
Ergothioneine concentrations on day 0. Day 0 bloodstains were created by dripping fresh blood onto filter paper, and metabolite extraction was performed immediately after bloodstain production. (**A**) Comparison of ergothioneine concentrations between the youth and elderly groups on day 0. The youth group is represented by the blue bars and the elderly group is represented by the red bars. (**B**,**C**) Comparison of ergothioneine concentrations between the youth group and the elderly group in sex-separated groups. (**D**,**E**) Comparison of ergothioneine concentrations between males and females in the same age group. The male group is shown in light blue, the female group is shown in pink. An unpaired *t*-test or Mann–Whitney test was performed for comparison between groups, and the values in the graph represent Mean ± SD. ** *p* < 0.01 and **** *p* < 0.0001.

When all participants were classified into youth and elderly groups for the day 0 bloodstain, reflects fresh blood status, the difference in ergothioneine concentration between them was statistically significant (Figure 1A). Additionally, when the participants were grouped by sex, the ergothioneine concentrations in day 0 bloodstains from elderly males and females tended to be higher than those from the youth group (Figure 1B,C). Furthermore, there were no differences in ergothioneine concentrations between male and female in the same age group (Figure 1D,E).

### 2.2. Changes in Ergothioneine Concentrations in Aged Bloodstains and Estimation of Age of Bloodstain Donors 

**Figure 2:**
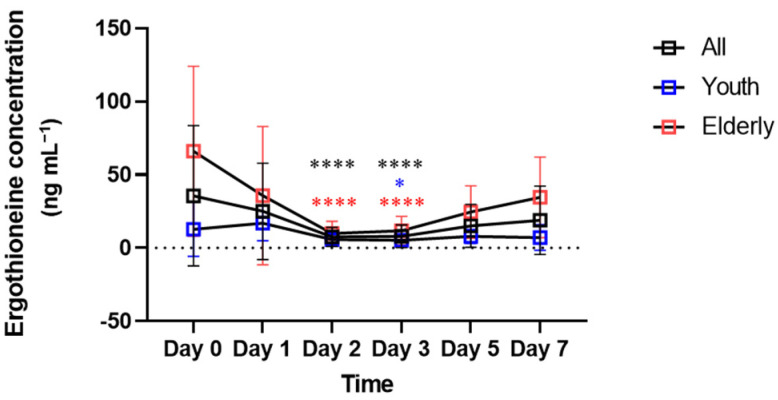
Ergothioneine concentrations in bloodstains over time. Mean and standard deviation are indicated by square and error bars. Black represents the entire subject, blue represents the youth group and red represents the elderly group. To compare the conditions on day 0, which reflects fresh blood, with those on other days, ANOVA or the Friedman test was performed. * *p* < 0.05 and **** *p* < 0.0001.

**Figure 3:**
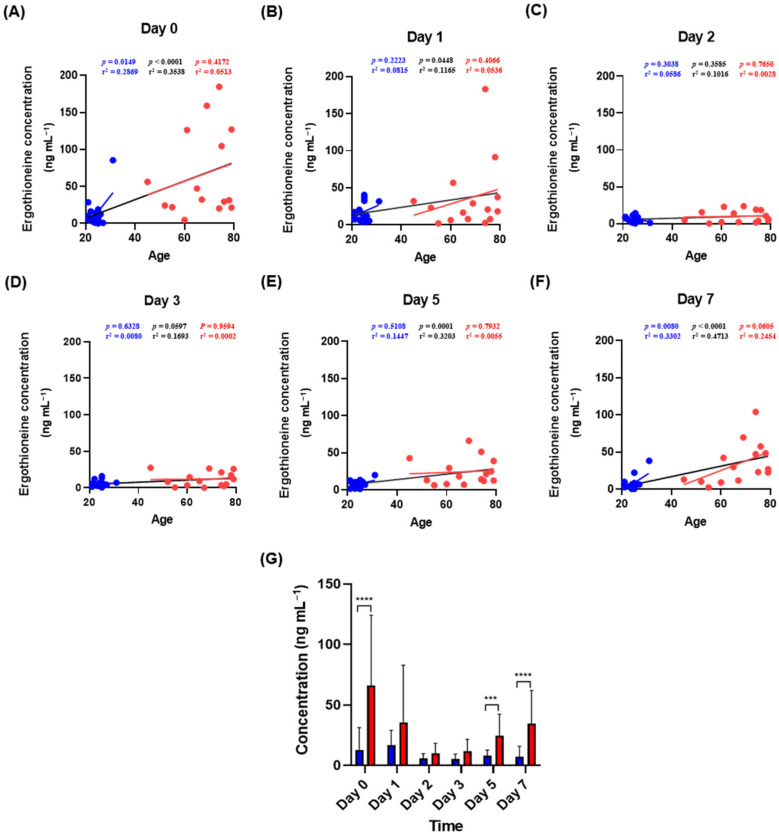
Relationship between donor age and ergothioneine concentration in bloodstains. (**A**–**F**) Correlation analysis of donor age and ergothioneine concentration at each time point. The *p*-value, r^2^ and trend line of the elderly (red), youth (blue) and the entire group (black) were calculated using Pearson or Spearman correlation analysis. Red dots represent elderly subjects, and blue dots represent youth subjects. (**G**) Age-divided significance within each time point. The youth group is represented by the blue bars and the elderly group is represented by the red bars. The *p*-values were calculated using unpaired *t*-tests or Mann–Whitney tests. Values in the graph represent Mean ± SD. *** *p* < 0.001 and **** *p* < 0.0001.

The effectiveness of ergothioneine as a marker was confirmed by subdividing the interval of 0–7 days by checkpoint at 0, 1, 2, 3, 5 and 7 days. In the overall pattern from days 0 to 7, no clear increase or decrease was observed over time (Figure 2). Statistical analysis of 15 combinations, comparing whether the concentration of ergothioneine between each point in time was significantly different, confirmed a significant difference between days 2 and 3 from that on day 0. This result was also confirmed in the elderly group, whereas only day 3 showed a significant difference from day 0 in the youth group. Figure 3 shows the correlation between ergothioneine concentration in the bloodstain and donor age. Except for days 2 and 3, the ergothioneine concentrations in the bloodstains increased with donor age (Figure 3A,B,E,F). The differences in ergothioneine concentrations between the youth and elderly groups on days 0, 5 and 7 were statistically significant (Figure 3G). 

### 2.3. Time-Dependent Changes in the Concentrations of Histidine and Hercynine in Bloodstains

**Figure 4:**
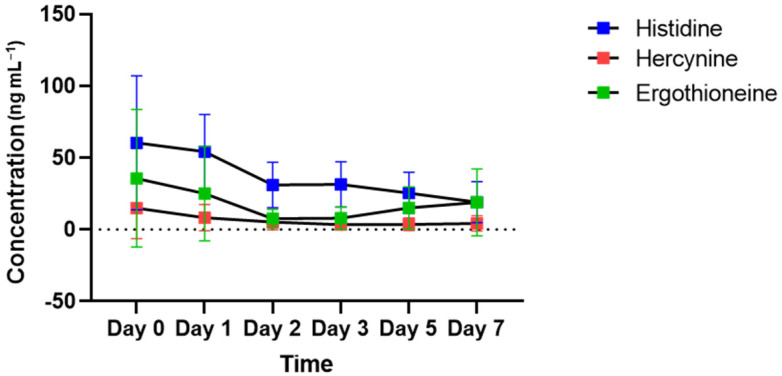
Time-dependent changes in the concentration of metabolites for the ergothioneine biosynthesis pathway in all subjects. Histidine (a precursor of ergothioneine) is indicated in blue, hercynine (produced from histidine) is indicated in red and ergothioneine (synthesized from hercynine) is indicated in green.

Figure 4 shows the changes in the concentrations of histidine, hercynine and ergothioneine present in the ergothioneine biosynthesis pathway in bloodstains left in vitro. Histidine concentrations show a gradual decrease from day 0 to day 7. The concentration of hercynine that can be produced from histidine in ergothioneine-producing organisms gradually decreased until day 2 and remained at that level until day 7. Ergothioneine, which can be generated from hercynine, decreased until day 2 and showed a tendency to increase after day 3. The trend from day 3 to day 7 was reversed for histidine.

## 3. Discussion

Ergothioneine, a histidine-derived thiol/thione [29,30], is naturally synthesized by microorganisms such as fungi and bacteria. There is no direct evidence of biosynthesis in animals and higher plants, but it is present in the cells and tissues of most plants and animals [29]. Ergothioneine is a colorless, odorless compound with a mass of 229.30 [29]. In humans, ergothioneine is acquired through diet [31,32,33,34] and accumulates in high concentrations in RBCs, bone marrow and liver via transporters such as OCTN1 [30]. As ergothioneine exists in the form of thione rather than thiol at physiological pH, it is stable and undergoes oxidation at a slower pace than simple thiol [29]. Therefore, ergothioneine is generally known as an antioxidant that protects tissues from oxidative stress [35,36,37]. The relationship between ergothioneine concentrations and age has been evaluated in several studies. In a sample of males in Saudi Arabia, ergothioneine concentrations in RBCs increased rapidly from age 11 to 18, peaked, gradually decreased after the age of 19 and remained constant until the age of 50 [38]. In a subject group recruited from an area surrounding the Jurong community in Singapore, the plasma ergothioneine concentration decreased with age in individuals above 60 years of age [39]. This decrease may be caused by several factors such as diet, increased turnover and changes in the transport function of the OCTN1 transporter [30]. 

Several studies have reported differences in metabolites depending on the sex and age of subjects [40,41,42]. Although the study consisted of a relatively small sample of 35 participants, there was no difference in sex distribution between the youth and elderly groups (Table 1). In a previous study, the ergothioneine concentration in bloodstain samples stored at room temperature for up to 4 h was significantly higher in the elderly group than in the youth group [28]. In this study, the ergothioneine concentration in the day 0 bloodstains showed a similar trend (Figure 1A). The concentration of ergothioneine in bloodstains was compared by placing participants between the ages of 21 to 31 years in the youth group and those between the ages of 45 to 79 years in the elderly group (Table 1). Another study used a similar grouping to classify the age of bloodstain donors [28]. The results of these two studies confirmed that ergothioneine was present at higher concentrations in the blood of the elderly group than in that of the youth group. This can be attributed to an increase in the accumulation of ergothioneine in RBCs with aging [38]. In addition, the results of this study, in which there was no significant difference between the sexes in the youth and elderly groups, support the results of previous studies, confirming that there was no difference in serum ergothioneine concentration between men and women aged 55 years or older [43].

In an in vitro environment, bloodstains undergo biochemical changes. During this process, environmental factors, such as light, temperature, humidity and microorganisms, as well as the surface on which the bloodstains are present and the contaminants that may mix with the bloodstains, can affect these biological processes. Observable and measurable morphological and biochemical changes in bloodstains can be used as indicators for estimating the age of the bloodstains [1]. In a previous study, mass spectrometry of bloodstain metabolites on days 0, 7, 14 and 21 revealed that ergothioneine levels decreased rapidly from day 0 to day 7 and gradually decreased to near non-existent levels from day 7 to day 21. Therefore, ergothioneine is considered a useful marker for estimating whether bloodstains are older than 21 days [4]. However, in this study, a significant difference between days 0 and 3 was observed for all subjects in the youth and elderly groups. This confirmed the change in ergothioneine concentration in the range of bloodstain ages when those ages are subdivided into smaller units than in previous studies [4]. Therefore, ergothioneine concentrations can potentially be used as a marker of elapsed time since deposition of the bloodstain for a limited period.

**Figure 5:**
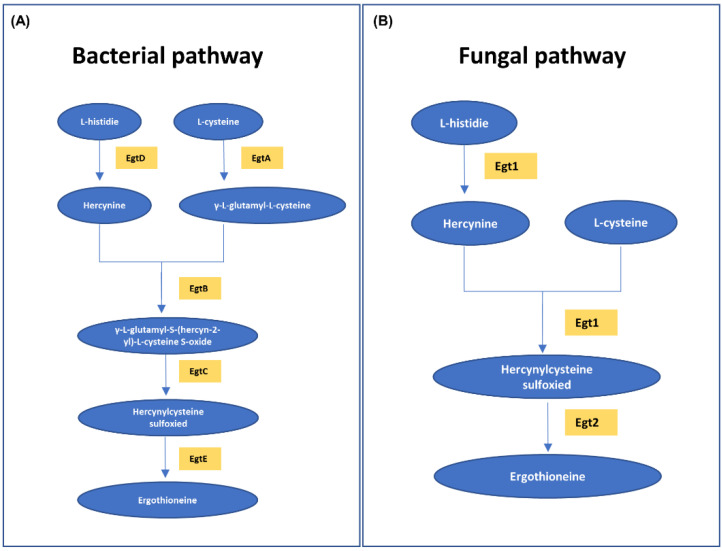
Ergothioneine biosynthesis. (**A**) Ergothioneine biosynthesis in bacteria and (**B**) ergothioneine biosynthesis in fungi via the MetaCyc pathway. The pathways are represented by the substances (blue circles) and enzymes (yellow squares) involved.

There is a difference between the in vivo metabolism of ergothioneine (abundant in the RBCs) and the biochemical reaction in the in vitro environment. As blood outside the body is exposed to various environmental factors, unpredictable biochemical reactions may occur in bloodstains, which distinguish the bloodstains from freshly acquired whole blood, blood cells and plasma (serum), and the concentration of ergothioneine in the bloodstains may change accordingly. If these changes in the ergothioneine concentrations in bloodstains can be determined by focusing on the difference between in vivo metabolism and in vitro biochemical reactions, it is possible to obtain useful information for crime scene reconstruction. In vivo, ergothioneine is obtained only through diet [34] and biosynthesized from histidine amino acids in fungi, such as mushrooms, or in bacteria [34,44,45,46]. Bloodstains after day 0 are affected by microorganisms in the external environment. Figure 5 shows ergothioneine biosynthesis. The enzyme involved in the process of synthesizing ergothioneine from histidine exists in fungi and bacteria. The biosynthesis of ergothioneine in microorganisms (Figure 5A,B) consists of methylating L-histidine to hercynine and adding sulfur derived from cysteine [46]. The changes observed in ergothioneine concentrations depending on the age of the bloodstains were not constant. It is possible that the decrease caused by the oxidation of ergothioneine exposed to the in vitro environment was greater than the increase caused by microbial synthesis at the beginning when the blood was fresh. The reason for the decrease in ergothioneine concentrations from days 1 to 3 is presumed to be oxidative degradation. However, the oxidative degradation of ergothioneine to the end product hercynine within 3 d has been reported only in the physiological environment of the body [47]; there are no reports on the external environment which has completely different physicochemical conditions from the internal environment of the human body. Moreover, as materials that act as oxidizing agents of ergothioneine in the external environment are not known, we can assume that ergothioneine is oxidized to hercynine by day 3. Furthermore, the amount of ergothioneine produced was greater than the amount oxidized after day 3. In addition, the decrease in histidine concentrations and increase in ergothioneine concentrations after day 3 showed opposite tendencies (Figure 4). Therefore, the possibility of ergothioneine biosynthesis by microorganisms cannot be completely eliminated. In the future, experiments on ergothioneine oxidative degradation in both internal and external environments should be conducted.

The concentrations of ergothioneine in the day 0, 5 and 7 bloodstains were higher for the elderly group than for the youth group (Figure 3G). The increase in the ergothioneine concentration according to the age of the bloodstains obtained from the elderly group after day 3 (Figure 2) may be related to the concentration of histidine, a precursor of ergothioneine, in addition to the effect of microorganisms capable of ergothioneine biosynthesis. Using plasma kinetics analysis, a previous study confirmed that the levels of essential amino acids in the blood 60–200 min after oral intake of essential amino acids, including histidine, was higher in elderly individuals than in young individuals [48]. These results suggest that histidine may be present in higher amounts in the blood of the elderly group than in the blood of the youth group. Therefore, as ergothioneine concentrations increase in RBCs because of accumulation owing to aging, microbial biosynthesis and histidine absorption rates, it is possible that ergothioneine may be present at higher levels in the elderly group than in the youth group. Hence, it may be used as an age-distinguishing marker of the bloodstain donor.

## 4. Materials and Methods

### 4.1. Chemicals and Reagents

High performance liquid chromatography-grade methanol, water and acetonitrile were purchased from J.T. Baker (Phillipsburg, NJ, USA). Formic acid (mass-spectrometry grade) was obtained from Fluka Analytical (Buchs, Switzerland) and ergothioneine standard was from AbaChemScene (Monmouth Junction, NJ, USA). L-Histidine standard was purchased from Sigma-Aldrich (St. Louis, MO, USA). L-Hercynine, L-Hercynine-d3 and ergothioneine-d9 were purchased from Toronto Research Chemicals (Toronto, ON, Canada) and DL-histidine-d3 was procured from Cayman (Ann Arbor, MI, USA). Deuterium-labeled standards, DL-histidine-d3, ergothioneine-d9 and L-Hercynine-d3 were used as internal standards in MS analysis. The reproducibility of the MS analysis was confirmed using quality control (QC) samples prepared in mobile phase A; the concentration of the internal standard for QC1 was 75 ng mL^−1^ and that for QC2 was 125 ng mL^−1^ (Supplementary Materials: Table S1).

### 4.2. Sample Collection

**Figure 6:**
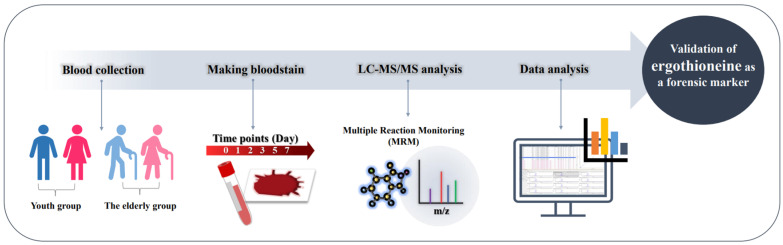
Experimental workflow. Bloodstains were produced with blood obtained from youth and elderly group individuals, and then metabolite extraction and target quantification were performed.

Blood samples were prepared by obtaining venous blood from 35 participants. All participants fasted for more than 8 h before blood collection. There were 20 individuals in the youth group, 15 individuals in the elderly group and a total of 14 males and 21 females in both groups. Eleven patients in the elderly group were known to have underlying diseases. Blood was collected using a syringe without any anticoagulant or other adducts. Demographic information of the subjects is summarized in Table 1, and the overall experimental workflow is schematically shown in Figure 6.

### 4.3. Sample Preparation 

Day 0 bloodstains were formed by directly dripping fresh blood onto filter paper, and metabolites were extracted immediately after bloodstains were created. Thus, the day 0 bloodstains reflect fresh blood. The stain samples were stored away from light in a room at a temperature of 22.9 ± 1.1 °C and relative humidity of 64.9 ± 10.9% for up to 7 days. Metabolites were extracted from the bloodstains on days 1, 2, 3, 5 and 7.

To extract the metabolites from the bloodstains, the bloodstain sample was cut into 16 pieces and transferred to a 1.5-mL tube. Next, 200 µL of HPLC-grade water was added and the mixture was vortexed for 30 s. Thereafter, 600 µL of 100% methanol, stored at −70 °C, was added. The mixture was vortexed briefly and stored in a deep freezer at −70 °C for 30 min for quenching. After quenching, the samples were vortexed for 1 min, centrifuged for 10 min at 14,000× g at 4 °C, sonicated for 10 min and allowed to stand at room temperature for 10 min. All centrifugation processes mentioned above were performed at 4 °C and 14,000× g. After centrifugation, the supernatant was transferred to another 1.5-mL tube. The same procedure for obtaining the supernatant was repeated thrice by adding 80% methanol to the remaining bloodstain. The obtained supernatant was completely dried using a vacuum concentrator (Scan Vac, LaboGene, Lynge, Denmark).

To obtain the metabolites, a filtering process to remove as many impurities as possible was performed on the dried supernatant, using a Nanosep^®^ Centrifugal Device with an Omega™ Membrane-3K (Pall Corporation, Port Washington, NY, USA). Before use, the filter was activated using water and 70% ethanol. The completely dried sample was dissolved in 500 µL of 80% methanol and vortexed, sonicated, allowed to stand at room temperature and centrifuged for 10 min at 14,000× g at 4 °C for each step. Next, the sample solution, except for the pellet, was transferred to a filter and centrifuged for 20 min at 14,000× g at 4 °C, and the filtrate was transferred to a new tube. Thereafter, 150 µL of water was added to the remaining samples in the filter, followed by centrifugation for 5 min at 14,000× g at 4 °C. Samples, in addition to the previously transferred sample solution, were then completely dried using a vacuum concentrator. For LC-MS/MS analysis, 80 µL of 5% acetonitrile and 0.1% formic acid solutions in water were mixed with the dried sample. After 10 min each of vortexing, sonication, standing at room temperature and centrifugation, the supernatant was transferred to a tube for long-term storage. Thereafter, the sample was diluted 200-fold with 95% HPLC water, 5% acetonitrile and 0.1% formic acid solution in a mass vial.

### 4.4. Quantification of Ergothioneine

For multiple reaction monitoring, compound optimization was first performed to obtain running parameter values. General standards and isotopically labeled standards were prepared as a 0.1-μg/mL solution and injected into the QTRAP 5500 mass spectrometer (AB Sciex, Framingham, MA, USA) using a syringe. Through compound optimization, collision energy, declustering potential, collision exit potential and entrance potential values were obtained, and the method was established (Table 2). To prepare the calibration solution, first, a high-concentration stock containing each general standard and isotopically labeled standard at 10 μg/mL was prepared in HPLC-grade water. Then, by diluting the general standards in HPLC-grade water, nine calibration solutions were prepared: 5, 10, 50, 100, 150, 200, 250 and 500 ng/mL. The calibration solutions were also added so that the isotope-labeled standards were present at 50 ng/mL each as internal standards. The metabolites in the samples were quantified using the concentration calculation formula of the calibration curve. A QTRAP 5500 mass spectrometer (AB Sciex) was used for target quantification of ergothioneine in the bloodstain. Agilent ZORBAX Eclipse Plus C18 (2.1 mm × 50 mm, 1.8 μm) was used as the analysis column, and Agilent ZORBAX StableBond-C8 (2.1 mm × 5 mm, 1.8 μm) (Agilent, Santa Clara, CA, USA) was used as the guard column to protect the analysis column. Mobile phase A comprised water containing 0.1% formic acid and 5% acetonitrile, and mobile phase B was composed of acetonitrile containing 0.1% formic acid and 5% water. The flow rate of the mobile phase was 400 μL/min. The gradient started with 100% A, decreased to 0% at 5 min and was maintained at 100% A from 7 to 10 min. Analyst Software version 1.6.1 (AB Sciex) was used for data processing. MultiQuant Software version 2.0.2 (AB Sciex) was applied to calculate the corresponding peaks of multiple reaction monitoring signals. 

### 4.5. Data Analysis

After quantitative results were obtained, statistical analysis and artwork illustration were performed using GraphPad Prism software (version 8.4.2, San Diego, CA, USA). The normality of the data was tested using the Shapiro–Wilk test. On day 0, the Mann–Whitney test was used to compare ergothioneine concentrations between groups according to age and sex (Figure 1). A repeated-measures ANOVA or Friedman test was used to compare the ergothioneine concentrations among the six bloodstain age conditions (Figure 2). In addition, the correlation between age and ergothioneine was analyzed using correlation analysis and linear regression analysis (Figure 3A–F). Unpaired *t*-tests with Welch’s correction and Mann–Whitney tests were used to compare ergothioneine concentrations between youth and elderly at the same point in time (Figure 3G). The MetaCyc [31] open database was used for the pathway analysis. 

## 5. Conclusions

The mean concentration of ergothioneine in bloodstains over 7 days was higher in the elderly participants than in the younger ones. On days 0, 5 and 7, the difference in the bloodstain ergothioneine concentrations between the youth and elderly groups was statistically significant, confirming that age classification was possible. In addition, the changes in the ergothioneine concentration with the age of the bloodstain showed a statistically significant difference on days 2 and 3 compared to day 0. The results confirmed the potential use of ergothioneine as a marker to estimate the age of the bloodstain donor and that of the bloodstain. Follow-up studies that confirm the relationship between the ergothioneine concentrations in the bloodstain, the amount of precursor present in the source blood and the biosynthesis of ergothioneine in the in vitro environment would improve the applicability of the ergothioneine present in the bloodstain as a marker in forensic science.

## Figures and Tables

**Table 1:** Demographic information of subject group.

Group	Youth	Elderly	*p*-Value
	(*n* = 20)	(*n* = 15)	
Age (mean ± standard deviation)	24 ± 2	67 ± 11	*p* < 0.05 ^a^
Sex (male/female)	9/11	5/10	0.486 ^b^
Disease	0	11	
Smoking	2	1	
Fasting	>8 h		

^a^ Mann–Whitney test, ^b^ Chi-square test.

**Table 2:** Multiple reaction monitoring transition parameters.

Analytes (Quantifier/Qualifier)	Q1 (Mass, Da)	Q3 (Mass, Da)	DP (V)	CE (eV)	EP (V)	CXP (V)
Ergothioneine (quantifier)	230.066	186.100	46	17	10	12
Ergothioneine (qualifier)	230.066	127.100	206	25	10	12
Ergothioneine-d9 (quantifier)	239.124	127.100	106	27	10	10
Ergothioneine-d9 (qualifier)	239.124	195.200	106	17	10	12
L-Hercynine (quantifier)	197.957	95.100	61	27	10	4
L-Hercynine (qualifier)	197.957	60.100	16	19	10	4
L-Hercynine-d3 (quantifier)	200.770	95.100	71	29	10	8
L-Hercynine-d3 (qualifier)	200.770	63.200	71	21	10	8
L-Histidine (quantifier)	155.940	110.100	61	19	10	16
L-Histidine (qualifier)	155.940	56.100	61	43	10	4
DL-Histidine-d3 (quantifier)	158.878	113.200	56	19	10	6
DL-Histidine-d3 (qualifier)	158.878	57.100	56	27	10	10

Collision energy (CE), declustering potential (DP), collision exit potential (CXP) and entrance potential (EP).

## Data Availability

Data will be made available on request.

## Supplementary Materials

The following supporting information can be downloaded at: https://www.mdpi.com/article/10.3390/molecules27248885/s1, Table S1: Determining the analytical reproducibility with quality control sample.
