# Supplementary material for: Validation of the Metabolite Ergothioneine as a Forensic Marker in Bloodstains
Source: Molecules. 2022 Dec 14;27(24):8885. doi: 10.3390/molecules27248885 (PMC9786767; doi:10.3390/molecules27248885)
Supplement: Supplementary file 1 [file molecules-27-08885-s001.zip › molecules-2045573-supplementary.pdf]

Validation of the Metabolite Ergothioneine as a Forensic Marker in Bloodstains

Seungyeon Lee[1], Sora Mun[2], You-Rim Lee[1], Jiyeong Lee[2,*] and Hee-Gyoo Kang[1,2,*]

1 Department of Senior Healthcare, Graduate School, Eulji University, Uijeongbu 11759, Republic of Korea

2 Department of Biomedical Laboratory Science, College of Health Sciences, Eulji University,

Seongnam 13135, Republic of Korea

3 Department of Biomedical Laboratory Science, College of Health Science, Eulji University,

Uijeongbu 11759, Republic of Korea

* Correspondence: leejiyeong@eulji.ac.kr (J.L.); kanghg@eulji.ac.kr (H.-G.K.);

Tel.: +82-31-951-3862 (J.L.); +82-31-740-7315 (H.-G.K.)

Table S1. Determining the analytical reproducibility with quality control sample

Metabolite	Quality control sample	Mean	Standard deviation	Percent coefficient of variation
Ergothioneine	QC1 (75 ng/mL)	62.9	3.5	5.5
	QC2 (125 ng/mL)	111.0	8.4	7.5
Hercynine	QC1 (75 ng/mL)	73.4	7.8	10.6
	QC2 (125 ng/mL)	123.6	15.1	12.2
Histidine	QC1 (75 ng/mL)	14.0	1.7	12.1
	QC2 (125 ng/mL)	24.9	2.5	10.1
